# Health taxation and need to pay health related taxes in Iran: A scoping review

**DOI:** 10.34172/hpp.025.44434

**Published:** 2025-12-30

**Authors:** Hossein Dargahi, Mahdi Kooshkebaghi, Nasrin Abolhasanbeigi Gallehzan

**Affiliations:** ^1^Department of Health Management, Policy, and Economics, School of Public Health, Health Information Management Research Center, Tehran University of Medical Sciences, Tehran, Iran; ^2^Department of Health Management, Policy, and Economics, School of Public Health, Tehran University of Medical Sciences, Tehran, Iran; ^3^Health Management and Economics Research Center, Health Management Research Institute, Iran University of Medical Sciences, Tehran, Iran

**Keywords:** Health care system, Iran, Scoping review, Taxation

## Abstract

**Background:**

Health care taxation is one of the government’s income sources. In Iran, despite the high consumption of harmful products, there are limitations in healthcare taxation, which may be due to the high wall of distrust between the government and taxpayers. In this study, we aimed to identify and analyze the reasons for paying health care taxes in developed countries in comparison with Iran.

**Methods:**

In this scoping review, the data were collected among the evidence published from 2000 to 2022 in English and Persian. A search strategy was conducted to find all the evidence and resources published using the selected keywords across several databases. Finally, 129 articles were confirmed to be included in the review.

**Results:**

Taxes on harmful goods, including sin taxes value, added taxes, and green taxes were among the most important and sustainable resources to improve the health care system in each country. In Iran, despite the approval of various taxes on harmful goods and polluting industries for public health reasons, these policies have not yet been implemented.

**Conclusion:**

For Iran, we recommend strictly implementing the tax policies, with allocations tailored to local conditions, to reduce the financial burden on the Iranian healthcare system.

## Introduction

 As common place notion, economic growth leads to development, and may directly impact on well-being and quality of life.^1^ In recent decades, healthcare costs have been increased in all countries.^2^ Tax is one of income sources for governments,^3^ which means that the higher is the share of taxes in financing government expenditures, the less may be the possibility of adverse economic effects.^4^ In any country, tax planning is a legitimate method for mitigating adverse economic effects, as it enhances a company’s attractiveness to creditors and investors by ensuring stability and long-term solvency.^5^

 The World Health Organization (WHO) has recommended the use of tax instruments and innovative projects to promote health systems in low and middle countries.^6^ Health taxes (excise taxes on tobacco, alcohol, and sugar-sweetened beverages) are examples of important tools to improve health and fiscal outcomes associated to public income budgeting and treasury.^7^ The introduction or reform of health taxes can strengthen the healthcare system by curbing the consumption of health-harming commodities and their consequent negative effects.^8^ Such efforts can also improve fiscal balances by increasing tax revenue and reducing health care costs associated with illness and injuries in the long run.^9^

 Taxes are powerful tools for reducing the consumption of unhealthy products, creating financial sustainability, and potential capacity of the health systems in a country.^10^ Fichera found that increasing the taxes on tobacco, including cigarettes, could significantly reduce the harmful effects of smoking and save the lost revenue.^11^ A 2021 study in South Africa declared that taxing on sugary drinks could prevent 72,000 deaths and 550,000 years of healthy life lost due to stroke, and also save $5 billion in healthcare costs.^12^ In addition, various taxes, such as value-added tax and green taxes, may directly affect the financing process of health care systems.^13^ Value Added Tax (VAT) is incorporated into a chain of economic transactions. Standard practice requires firms to become VAT-obligated once their turnover exceeds a specific threshold, compelling them to charge tax on their sales but allowing them to reclaim the tax paid on their inputs.^14^ The collection of specific tax incomes should have a specific use, and the priority of their use should be clear.^15^

 According to a WHO report, many countries use revenues from specific taxes to improve their health care systems.^16^ The tax rate on goods harmful to health should be set to generate significant revenue while safeguarding the minimum welfare of households.^17^ Torki et al showed that a 10% tax increase on soft drinks may reduce their consumption by 11.4%.^18^ So, the tax increase leads to a decrease in the consumption of these types of sugary drinks due to the increase in price.^19^

 Fattahi et al found that the higher VAT rates on cigarette may be significantly associated with reduced levels of cigarette smoking.^20^ Feizpour et al also found that despite the high prevalence of tobacco and alcohol use in the target population, excessive exposure to unhealthy product advertising was associated with increased public support for media regulation.^21^ In fact, there is a great need to understand the factors that may influence taxpayers’ perceptions, personal values, and personal tax culture. Previous reward by the Organization for Economic Cooperation and Development (OECD) did not provide a sufficient explanation of all the socio-economic factors associated with personal tax culture.^22^ It is also necessary for taxpayers to understand when to propose tax moral incentives within the institutional pillars.^23^ Khoshakhlagh et al reported that sugar-sweetened beverage industry resistance can persist after enacting new policies, as vested interests may seek to resist legislation, and thus block or prevent the introduction of tax policies.^24^ In another study, Amiry et al reported that taxes alone may not reduce the burden of diet-related diseases, but they can significantly change the behavior of industry, producers, and consumers.^25^ Besides, Hofman et al declared that the beverage industry opposed the imposition of tax on sugar-sweetened beverages, which may be due to economic harm, unemployment of associated workers, and any possible harm to small businesses in the United States and Latin America.^26^ Therefore, taxing harmful products might change consumer behavior and, thus, the optimal taxation on harmful goods may have a dual benefit of improving not only public health, but also economic performance and social welfare.^27,28^

 The most important type of tax addressed in the new century to improve the healthcare system is the green tax, which is essential for controlling environmental pollution and protecting the environment.^29^ Gholami and Mousavi showed the effects of air pollution on healthcare indicators, and reported that the environmental taxes may increase the welfare of individuals in a society.^30^ In addition, green tax reform may improve local air quality and reduce the levels of air pollution in neighboring cities and countries.^31^ The results of the study performed by Moghimi et al also showed that policymakers should achieve pollutant reduction through raising tax rates and strengthening tax exemptions to encourage companies.^32^

 In Iran, despite the high consumption of harmful products, the tax rate has not yet been increased, considerably. For example, tobacco taxes are only 21.7% of the retail price.^33^ Despite the efforts made in Iran, the current state of tax paying for health care system is problematic, which may be due to the high wall of distrust between the government and the tax payers.^34^ In low-income countries like Iran, the tax system seems to work better when there is a high rate of formal employment, despite issues of unfair wealth redistribution.^35^ In the health system of such countries, the following efforts may help in improving the implementation of the tax system: the designation of comprehensive tax information, customer communication management, classification of tax payers establishment of vital tax system, comprehensive training courses for tax office’ employees, establishment of modern tax paying basis, cleaning of economic system, considering the tax equity, and preventing tax evasion

 The application of a green tax on pollutants yields a net positive welfare effect due to the benefits of reduced pollution, and this effect intensifies as the tax rate increases. Consequently, there is no doubt about the merit of implementing green taxes in Iran. One study identifies a 4 percent tax as the optimal rate, resulting in the highest welfare growth.^36^ Simultaneously, the introduction of VAT represents a modern tax base. For it to support healthcare programs and objectives in Iran effectively, its efficient establishment must be accompanied by the development of a robust tax culture and increased taxpayer satisfaction.^37^ Finally, improving the tax system related to the Iranian health sector requires a comprehensive strategy. Key measures include designing integrated tax information systems, implementing customer communication management, classifying taxpayers, establishing a vital records system, providing comprehensive training for tax office employees, creating modern payment platforms, cleansing the economic system, ensuring tax equity, and preventing tax evasion.^38^ In the present study, we aimed to identify and analyze the reasons for paying health care taxes in developed countries in comparison with Iran.

## Methods

 In this scoping review, we tried to identify the concepts, theories, sources of evidence, and gaps in scientific knowledge^39^ on health taxation. So, our objectives were:

 To identify and categorize types of health taxes and to analyze the current state of health-related tax systems in Iran and other countries.

 To identify and analyze the rationales for implementing taxes to fund the healthcare system.

 The study environment included the databases related to health system and health economics (Table 1).Moreover, Google Scholar was used as a research engine for additional documents.

**Table 1:** Databases and data banks

**Database name**	**Language**
1. Scientific Information Database (SID) 2. Iranian Research Institute for Information Science and Technology (IranDoc) 3. Indexing articles published in Iran Medical Journals (Iranmedex) 4. Magiran 5. Civilica (Publisher of Iran conferences and scientific journal papers) 6. Elmnet	Persian
1. PubMed 2. Science Direct 3. Scopus 4. ProQuest 5. Google Scholar	English

 The inclusion criteria were all original articles, reviews, letters, editorials, conference papers, gray articles, and the articles that presented the core and dimensions of health taxes published from 2000 to 2022, in Persian and English languages. Exclusion criteria were the studies with no full-text availability, and the articles that are unrelated to the mentioned keywords. The English keywords used in this study were those presented in Table 2, which either were searched alone, or in combination with Boolean operators AND and OR.

**Table 2:** Keywords used and reviewed by Boolean operators

**Used keywords**
1. Tax or taxation and healthcare or healthcare system
2. Specific tax and healthcare or healthcare system and Iran
3. Specific tax and green tax or healthcare system or Iran
4. Specific tax and value added taxes or healthcare system or Iran
5. Sale tax and taxation or green tax or value added taxes or healthcare
6. Harmful tax(es)/taxation
7. Taxes income and fiscal space or healthcare system and Iran
8. Tax culture and tax fiscal space, healthcare, or Iran
9. Unwealthy food and sugar-sweetened beverage consumption, or health taxation, price
10. Health taxes and Iran or tax culture, or tax evasion or tax system
11. Taxation and cigarette or beverage or sin tax
12. Taxation, and tax management or tax administration, and tax evasion, and health

 The included studies were examined independently by two authors. If there was no agreement between the two authors, a third author would help to resolve the disagreement. Tables 3 and 4 show the articles and the documents included in the study. The PRISMA-SCR (Preferred Reporting Items for Systematic reviews and Meta-Analyses extension for Scoping Reviews) was used to guide the process of study inclusion and reporting.

**Table 3:** Number of studies identified in Persian on health-related taxes

**Keyword**	**SID**	**Irandoc**	**Magiran**	**CIVILICA**
Tax	364	1352	1873	1289
Health tax(es)/taxation	2	4	13	3
Specific tax(es)/taxation	0	0	0	0
Health/Medical Tax	0	0	0	1
Harmful tax(es)/taxation	0	0	1	1
Unhealthy tax(es)/sin tax(es)	0	0	0	0
Cigarette tax(es)	0	0	0	0
Beverage tax	0	0	0	0
Drink tax(es)	0	2	1	0
Food tax(es)	0	0	2	0
VAT(s)/value added tax(es)	83	220	181	259
environmental tax(es)	13	28	24	29
Green tax(es)	21	35	30	61
Tax evasion/tax fraud	49	212	95	213
Tax management/tax administration	22	246	54	141

**Table 4:** Number of studies identified in English on health-related taxes

**Keyword**	**PubMed**	**Science Direct**	**ProQuest**
Tax	2705	4649	113639
Taxation	299	1046	34214
Health tax(es)/taxation	235	99	175
Specific tax(es)/taxation	3	23	29
Harmful tax(es)/taxation	72	3	137
Unhealthy tax(es)	83	8	4
Cigarette tax(es)	111	53	335
Beverage tax(es)	185	77	299
Drink tax(es)	59	33	103
Sin tax(es)	22	16	138
Food tax(es)	35	55	157
VAT(s)	183	833	14713
Value added tax(es)	23	28	3532
Environmental tax(es)	27	251	114
Green tax(es)	12	78	339
Tax evasion	29	194	3778
Tax fraud	9	9	442
Tax management	83	54	418
Tax administration	12	20	1998

## Results

**Figure 1:**
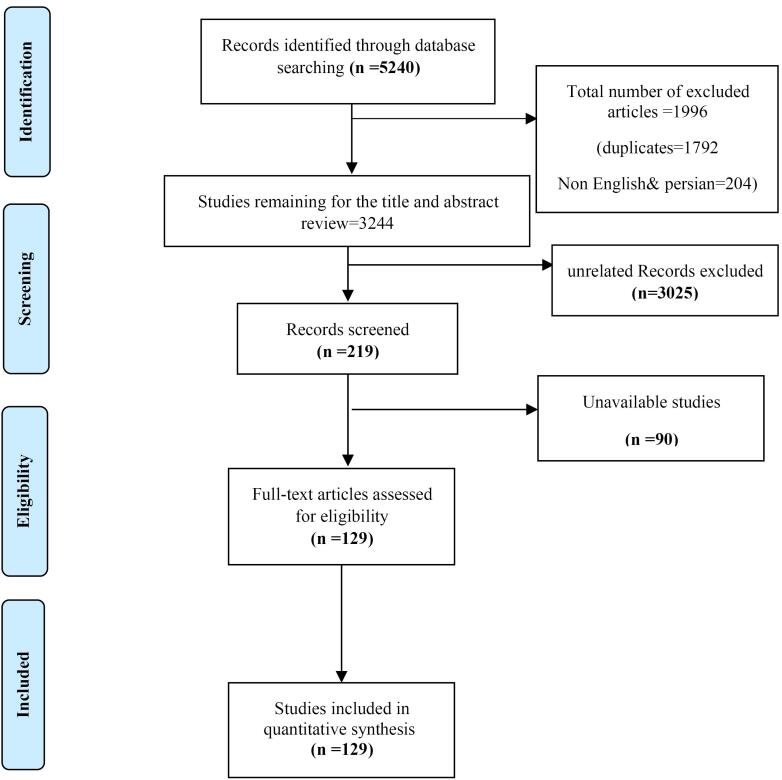
The PRISMA flow diagram for study process selection

 The initial database search with the relevant keywords resulted in 5240 articles, of which 1792were duplicates, and 204 were non-English and non-Persian publications. Therefore, 3244 articles remained for title and abstract review, within which 3025 articles were excluded, as they were unrelated records. Among the remaining articles (219), 90 articles were unavailable and so were removed. Finally, 59 articles in Persian and 70 articles in English (totally 129 studies) were included in the review (Figure 1).

 As presented in Tables S1 and S2, the most of the studies emphasized on implementing green tax, VAT and sin taxes as the preferable financial processes for health care systems, focusing on behavioral economics and tax evasion. Of course, the effects of health care taxation on Iranian economic growth and social welfare needs the Dynamic Stochastic General Equilibrium Approach (DSGE). In addition, several studies in developed and developing countries discussed on estimating the impacts of tax policy interventions on unhealthy foods and oral health, and how these policies may impact food purchases and response to sin taxes. Therefore, policy lessons from health care taxation should be considered according to gradual informs and teeth related benefits in each country.

Tables S3 and S4 showed the efficient impact on household consumption patterns to offset air pollution costs with environmental activities or pay more taxes. Furthermore, most studies in Persian and English concluded that taxes on harmful and unhealthy products yield dual advantages. These include improving societal well-being by reducing disease prevalence and modifying consumer behavior, as well as strengthening economic performance. The SWOT analysis of health care taxes (Tables S5 and S6) showed that there was no significant correlation between tax increase and social welfare. In other words, double benefit of health care taxation in Iran was not achieved because of the complexity of tax administration, lack of adequate and appropriate information, the weakness of appropriate software, and lack of adequate training of tax officials to implement the VAT system. We also found that in most developing and developed countries, increased health care taxation has not been even sufficient to reduce the prevalence of diabetes, nor is it projected to be in the future. Furthermore, industries use several tactics to oppose healthcare taxes, emphasizing the potential for negative economic impacts such as job losses and reduced performance. Therefore, despite various policy recommendations, the assessment of healthcare-related taxes often fails to incorporate distributive justice, which is essential for these policies to be equitable and beneficial.

## Discussion

###  Sin tax

 One of the most important taxes that significantly impacts on health care of each society is the tax on unhealthy products or sin tax.^40^ The proper implementation of tax allocation on harmful products improves not only health but also the economic performance of countries and the well-being of individuals in the society.^41^ Ghaderi et al reported that sin tax is a tool that, if properly implemented, can contribute to change customer behavior, and thus may have a positive impact on the health of populations.^42^ The results of the study conducted by Jabbari et al indicated that if the harmful goods tax was implemented correctly, it could promote consumer behavior and, as a result, positively impact the health of society.^43^

###  Value added tax 

 With increasing social sensitivity towards harmful goods, the appropriate rate of VAT should be increased to provide appropriate conditions for social welfare. In this regard, Askari et al suggested that different tax rates to be determined for luxury goods.^44^ However, Bovard et al reported that there may be a problem on the path of tax policy-making in the VAT system of Iran.^45^ VAT as a modern tax base can improve the health care system in Iran, if established efficiently.^46^ Paraje et al declared that VAT administration in Ethiopia has gassed different challenges including lack of awareness of taxpayers, resistance against registration for VAT by some traders, weak culture of text payers, and finally poor VAT administration,^47^ which is similar with the Iranian taxpaying system.

 Another study in Ethiopia on VAT administration, identified several key problems, including lack of sufficient number of skilled personnel and gaps in the administration process in the areas such as refunding, involving, and filling requirements,^48^ which is not compatible with the Iranian taxpaying experts and staffs. In Iran, national commitment and the creation of domestic tax culture were identified as the main factors for successful establishment of VAT paying system. Krass et al believed that sales promotion is often a kay component within company’s promotional complain, including a number of sales promotion techniques (SPTs). Although the use of SPTs is common, their VAT treatment is uncertain, which often creates a VAT cost. Therefore, the application of SPTs should be designed so that the burden falls not on businesses, but on consumers.^49^ Zeinali Ghasemi et al introduces the impacts of VAT on sales performance, which is a recently adopted forms of price promotion.^50^ SPTs and VAT - free promotion are the new techniques that can develop and promote the Iranian health taxation.

###  Green (environmental) tax 

 Iran lacks a state green tax; in fact, the government subsidizes fossil fuels. This leads to an uncontrolled increase in consumption, which not only depletes resources for future generations but also causes significant environmental damage. To address this, more serious measures are necessary to reduce consumption. Implementing a higher tax on oil, a lower tax on gas, and promoting solar energy would have a more favorable effect on public welfare and healthcare.^51^

 Zhang et al observed that innovation in China is still in a nascent stage, where market forces alone are insufficient for rapid development, necessitating government intervention. Their findings identified a U-shaped relationship between environmental regulation and green product innovation, indicating that the intensity of such regulations initially inhibits but eventually promotes innovation. Furthermore, R&D tax incentives were shown to significantly foster green product innovation.^52^ As an external benchmark, these results could inform the design of a health tax system in Iran.

 Furthermore, Noch and Rumasukun reported that implementing a green tax to prevent environmental pollution significantly reduces health-related costs by improving air quality. ^53^ Similarly, Drywa found that green taxes positively impact economic welfare and foster long-term economic growth.^54^ Supporting this, Gribnau and van Steenbergen’s research confirms the effectiveness of green taxes in regulating various aspects of environmental liability.^55,56^

###  Tax culture

 Although significant efforts have been made recently to improve people’s tax literacy, Iran’s tax system still lacks a strong tax culture. Unstable economic laws and regulations, the lack of appropriate solutions for the distribution of welfare goods, and the lack of welfare facilities for taxpayers have made Iran’s tax structure vulnerable, which may prevent the growth of tax culture in the country.^56^ Tax culture in Iran needs to be reformed based on the Iranian social culture. The increased attention to tax culture can be attributed to the elimination and reform of tax system laws in the countries like Iran. When trust and cooperation are internalized within a community, members have more incentive to support new tax policies and a greater willingness to pay taxes for public health improvements.^57^ The people’s trust in Iran to pay taxes is currently low, which may be due to their inappropriate economic situation, and the lack of transparency in spending and tax revenues.^58,59^

## Limitation

 As a scoping review, this study has inherent limitations. These may include the potential for bias in the included studies, a focus on breadth at the expense of depth, and constraints inherent to the methodological framework, such as fixed parameters that prevent scope creep. To rigorously address these limitations, the authors strictly adhered to the scoping review protocols outlined in the Joanna Briggs Institute (JBI) manual, ensuring a structured and transparent process.

## Conclusion

 This study has identified and analyzed various models of taxation—including its imposition, collection, and allocation—to propose a reformed framework for Iran’s healthcare system. Our study has also emphasized the challenges and weaknesses of the taxpay system and the lack of sufficient utilization taxpaying for allocation to healthcare and services in Iranian community

 Our analysis underscores significant challenges within the current Iranian tax system, particularly its weaknesses in efficiently collecting and allocating sufficient funds to healthcare services. We contend that health taxation in Iran requires profound reform. The existing laws, taxpayer incentive structures, and overarching strategic documents have failed to resolve the systemic issues that prevent adequate funding from reaching the public health sector. This failure may also be attributed to insufficient follow-up by key stakeholders, including the Iranian Ministry of Health.

 To foster a culture of tax compliance and social responsibility, we propose a multi-faceted strategy that includes launching transparent public campaigns on official social media to clearly link tax payments to specific healthcare benefits, implementing a tiered system of tangible incentives such as fast-tracked health services and public recognition certificates for timely taxpayers, and integrating fiscal citizenship education into public discourse. We predict that by demonstrating the direct value of contributions, offering meaningful rewards, and cultivating a sense of collective duty, this approach will significantly encourage voluntary compliance and reshape taxpayer behavior, thereby securing a more sustainable funding base for Iran’s healthcare system.

## Competing Interests

 The authors declare that they have no competing interests.

## Ethical Approval

 This article approved by Institutional Research Ethics Committee of School of Public Health & Allied Medical Sciences- Tehran University of Medical Sciences, Tehran, Iran, with the ethics code: IR.TUMS.SPH.REC.1398.332. Ethical issues (including plagiarism, informed consent, misconduct, data fabrication and/or falsification, etc) have been completely observed by authors.

## Supplementary files

Supplementary file 1 contains Tables S1-S6.
