# Supplementary material for: Health taxation and need to pay health related taxes in Iran: A scoping review
Source: Health Promot Perspect. 2025 Dec 30;15(4):325–32. doi: 10.34172/hpp.025.44434 (PMC13156257; doi:10.34172/hpp.025.44434)
Supplement: Supplementary file 1 — contains Tables S1-S6. [file hpp-15-325-s001.pdf]

Dargahi et al, **Health Promotion Perspectives**, 2025;15(4):325-S1
doi: 10.34172/hpp.025.44434
https://hpp.tbzmed.ac.ir

Supplementary file 1

Table S1: Total number of selected Persian articles on health-related taxes (2000 to 2022)

No	Author/s	Year	Country	Subject
1	Salem & gholami	2022	Iran	Estimating the Optimal Taxation Rate on Consumer Goods Harmful to Health by Microstimulating in Iran(1)
2	Rasmi et al.	2022	Iran	Feasibility Study of Implementing Green Tax System in Iran(2)
3	Farzinmehr et al.	2022	Iran	Evaluating the potential effect of tax policies on the consumption of soft drinks(3)
4	Sameti et al.	2021	Iran	Determining factors affecting tax evasion using meta-analysis method(4)
5	Choobineh et al.	2021	Iran	The Effect of Tax Evasion on Monetary Rule under Fiscal Dominance and Liquidity Constraint: The Case of Iran(5)
6	Homaie rad et al.	2020	Iran	Quality and quantity of price elasticity of cigarette in Iran(6)
7	Aghaei & Nahid	2004	Iran	Rate structure, justice and exemption in the value added tax system(7)
8	Sanaeepour	2020	Iran	Identifying and Prioritizing the Factors Influencing Tax Evasion in Small & Medium Enterprises (SMEs) from the Perspective of Iranian National Tax Administration Staff: A Mixed Method Study(8)
9	Nadri Khodabakhshi & Dahmardeh	2019	Iran	Investigating of the tax income and oil revenues on health expenditures in Iran(9)
10	Torki Harchegani	2018	Iran	Modeling the effects of green tax on health spending using a computable general equilibrium model(10)
11	gharibdosti & Ghasempanah	2019	Iran	The legal loopholes of tax evasion in Iran's financial laws(11)
12	Torki Harchegani & Dahmardeh	2017	Iran	Investigating the Effect of the Green Tax on Iran's Health Sector: A General Equilibrium Approach(12)
13	Torki Harchegani& Dahmardeh	2018	Iran	Simulating green tax effects on pollution reduction, mortality and morbidity costs in Iran(13)
14	Izadkhasti et al.	2017	Iran	Analyzing the Impact of Green Tax on Emission of Pollution and Health Index in Iran: A Simultaneous Equations Model(14)
15	Jabari & Ghazal	2016	Iran	Review of green tax and double benefit in Iran's economy(15)
16	Afshari et al.	2017	Iran	Tax evasion control mechanisms in Iranian law. MA thesis(16)
17	Keshtkaran et al.	2012	Iran	Value added tax in goods and services harmful to health(17)
18	Ahangari et al.	2018	Iran	effects of Green Tax on Economic Growth and Welfare in Economy of Iran: a Dynamic Stochastic General Equilibrium Approach (DSGE)(18)
19	Pakdaman et al.	2016	Iran	Explanation of Interaction between Iranian Physicians and Government in the Field of Tax: A Qualitative Study(19)
20	Jafari samimi et al.	2015	Iran	Application of the endogenous growth model to calculate the optimal value added tax rate with an emphasis on harmful and waste products(20)
21	Mobini et al.	2015	Iran	Identify barriers and drivers of establishment of VAT(21)
22	Etemad et al.	2016	Iran	The Status of Television Advertisements of Health-threatening Products(22)
23	Hosseinzadeh & Maddah	2018	Iran	The effect of pollution tax on houshould's demand for environment polluting goods(23)
24	Ziai Bigdeli et al.	2014	Iran	Identify barriers to optimal implementation of the VAT system in Iran(24)
25	Fattahi et al.	2016	Iran	Empirical Analysis of the relationship between air pollution and public health expenditures - A dynamic panel data approach(25)
26	Amiri M	2017	Iran	Behavioral economics and tax evasion(26)
27	Seyed Nourani & Amirshahi	2015	Iran	Two different rates on value added tax. Quarterly(27)
28	Mousavi Jahromi & Gholami	2015	Iran	Prediction of value added tax due to tobacco use in Iran using neural network method(28)
29	Khosh akhlagh et al.	2014	Iran	Green taxes and environmental standards of imports, appropriate tools of sustainable development in Iran's transition economy(29)
30	Feizpour et al.	2014	Iran	Green tax forgetting factor in Iranian industrial planning)(30)
31	Rousta & Heidarieh	2014	Iran	Ranking causes of tax evasion by Analytic Hierarchy Process (AHP) (31)
32	Hadian & Ostadzede	2013	Iran	Estimating the optimal level of pollution tax in Iranian economy(32)
33	Samadi & Tabandeh	2013	Iran	Tax Evasion in Iran: its Causes, Effects and Estimation. Journal of tax research(33)
34	Moosazadeh et al.	2013	Iran	Value added tax and its challenges. MA thesis. School of health public(34)
35	Hadian & Tahvili	2013	Iran	Tax Evasion and Its Determinants in the Iranian Economy (1971-2007) (35)
36	Mozayeni & Moradhasel	2012	Iran	Investigate the interactions between economic growth and environmental destruction on health(36)
37	Almasi et al.	2012	Iran	The Organizational Factors Affecting VAT Evasion (Case Study of Tehran City VAT Directorate) (37)
38	Milani et al.	2012	Iran	Tax Evasion from the Underground Economy in Iran(38)
39	Rashti & Milani	2011	Iran	Investigating the effect of VAT on income distribution in selected countries (39)

40	Moghimi et al.	2011	Iran	The Survey of Welfare and Environmental Effects of Green Tax and Decline Subsidy on Fueles in Iran by Using a Computable General Equilibrium Model(40)
41	Najarzadeh & Aghaei Khondabi	2011	Iran	Estimating the elasticity of demand for goods and services subject to consumption and sales tax in Iran(41)
42	Zehi & Mahammadkhani	2011	Iran	A Study on Factors Affecting Tax Evasion (A Case Study of East Azerbaijan Province) (42)

Table S2: Selected English articles on health taxes (2000-2022)

No	Author/s	Year	Country	Subject
1	**Siu & Thow**	**2022**	**WHO**	Linking health and finance ministries to improve taxes on unhealthy products(43)
2	Miracolo et al.	2021	Latim America	Sin taxes and their effect on consumption, revenue generation and health improvement: a systematic literature review in Latin America(44)
3	Hernández et al.	2021	Mexico	Taxes to Unhealthy Food and Beverages and Oral Health in Mexico: An Observational Study(45)
4	Tönnies et al.	2021	Germany	Estimating the impact of tax policy interventions on the projected number and prevalence of adults with type 2 diabetes in Germany between 2020 and 2040(46)
5	Hofman et al.	2021	South Africa	South Africa's Health Promotion Levy: Excise tax findings and equity potential(47)
6	Pedroza-Tobias et al.	2021	Mexico	Food and beverage industry interference in science and policy: efforts to block soda tax implementation in Mexico and prevent international diffusion(48)
7	Marinello et al.	2021	United States	Employment impacts of the San Francisco sugar-sweetened beverage tax 2 years after implementation(49)
8	Sacks et al.	2021	Australia	Do taxes on unhealthy foods and beverages influence food purchases? (50)
9	Fichera et al.	2021	Spania	How do consumers respond to "sin taxes"? New evidence from a tax on sugary drinks(51)
10	Li et al.	2021	China	Do environmental taxes reduce air pollution? Evidence from fossil-fuel power plants in China(52)
11	Wang et al.	2020	England	Optimal sin taxes in the presence of income taxes and health care(53)
12	Elliott et al.	2020	Australia	Health Taxes on Tobacco, Alcohol, Food and Drinks in Low- and Middle-Income Countries: A Scoping Review of Policy Content, Actors, Process and Context(54)
13	Summan et al.	2020	141, 166 and 176 countries	The potential global gains in health and revenue from increased taxation of tobacco, alcohol and sugar- sweetened beverages: a modelling analysis(55)
14	Rufael & Weldemeskel	2020	England	Do environmental taxes and environmental stringency policies reduce CO2 emissions? evidence from 7 emerging economies(56)
15	Guo et al.	2020	China	The effects of environmental tax reform on urban air pollution: A 16quasi-natural experiment based on the Environmental Protection Tax Law(57)
16	Wu et al.	2020	India	Impact of cigarette tax increase on health and financing outcomes in four Indian states(58)
17	Abdool Karim et al.	2020	South Africa	Industry strategies in the parliamentary process of adopting a sugar-sweetened beverage tax in South Africa: a systematic mapping(59)
18	Kao et al.	2020	Canada	The health and financial impacts of a sugary drink tax across different income groups in Canada(60)
19	Javadinasab et al.	2020	Iran	Comparing selected countries using sin tax policy in sustainable health financing: Implications for developing countries(61)
20	Luong & Vu	2020	Vietnam	Impacts of excise taxation on non-alcoholic beverage consumption in Vietnam(62)
21	Doble et al.	2020	Singapore	The effect of implicit and explicit taxes on the purchasing of 'high-in-calorie' products: A randomized controlled trial(63)
22	Bridge et al.	2019	England	A call to action: advocating for the integration of oral health promotion and public health via sugar sweetened beverage taxation(64)
23	Jain et al.	2019	England	Equity as a consideration in evaluations of health taxes: a systematic review(65)
24	Salgado & Ng	2019	Mexico	Understanding heterogeneity in price changes and firm responses to a national unhealthy food tax in Mexico(66)
25	Griffith et al.	2019	England	Tax design in the alcohol market(67)
26	Allcott et al.	2019	England	Regressive sin taxes, with an Application to the Optimal soda tax(68)
27	Partyka et al	2019	selected countries.	Sin tax as a public health tool-strengths and weaknesses of this economic solution(69)
28	Kim et al.	2019	Sout Korea	Public support for health taxes and media regulation of harmful products in South Korea(70)
29	Chaloupka	2019	United States	The Use of Excise Taxes to Reduce Tobacco, Alcohol, and Sugary Beverage Consumption(71)
30	Minh et al.	2018	Vietnam	Potential health impacts of increasing the cigarette tax in Viet Nam(72)
31	Wesseh & Lin	2018	China	Optimal carbon taxes for China and implications for power generation, welfare and the environment(73)
32	Shang et al.	2018	India	The Association between State Value-Added Taxes and Tobacco Use in India—Evidence From GATS and TCP India Survey(74)
33	Signal et al.	2018	New zealand	Appetite for health-related food taxes: New Zealand stakeholder views(75)
34	Springmann et al.	2018	England	Health-motivated taxes on red and processed meat: A modelling study on optimal tax levels and associated health impacts(76)

35	Smith et al.	2018	England	Should we tax unhealthy food and drink? (77)
36	Jensen et al.	2018	Denmark	State-of-the-art for food taxes to promote public health(78)
37	Du et al.	2018	South AFRICA/United States	Sugar-Sweetened Beverage Taxes: Industry Response and Tactics(79)
38	Tamir et al.	2018	Israeli	Taxation of sugar sweetened beverages and unhealthy foods: a qualitative study of key opinion leaders' views(80)
39	Nomaguchia et al.	2017	Australia	The impact on productivity of a hypothetical tax on sugar-sweetened beverages(81)
40	Pui & Othman	2017	Malaysia	Economics and environmental implications of fuel efficiency improvement in Malaysia: A computable general equilibrium approach(82)
41	Wright et al.	2017	Scatland	Policy lessons from health taxes: a systematic review of empirical studies(83)
42	Cobiac et al.	2017	Australia	Taxes and subsidies for improving diet and population health in Australia: A cost effectiveness modelling study(84)
43	Silver et al.	2017	United States	Changes in prices, sales, consumer spending, and beverage consumption one year after a tax on sugar-sweetened beverages in Berkeley, California, US: A before-and-after study (85)
44	Mccoy et al.	2017	England	Framing the tax and health nexus: a neglected aspect of public health concern(86)
45	Manyema et al.	2016	Sout Africa	Modelling the potential impact of a sugar-sweetened beverage tax on stroke mortality, costs and health-adjusted life years in South Africa(87)
46	Capacci et al.	2016	France	The impact of the French soda tax on prices, purchases and tastes: an ex post evaluation(88)
47	Hellowell et al.	2016	Scatland	Hard to avoid but difficult to sustain: Scotland's innovative health tax on large retailers selling tobacco and alcohol(89)
48	Briggs et al.	2016	England	Econometric and comparative risk assessment scenario modelling of the proposed UK sugary drink tax on health(90)
49	Reeves et al.	2015	England	Financing universal health coverage—effects of alternative tax structures on public health systems: cross-national modelling in 89 low-income and middle-income countries(91)
50	Hare & Curtis	2014	Malawi	Health spending, illicit financial flows and tax incentives in Malawi(92)
51	Paes et al.	2014	Brazil	Tax expenses and their impacts on performance in health and education(93)
52	Barry & Gollust	2013	United States	Taxes on Sugar-Sweetened Beverages: Results from a 2011 National Public Opinion Survey(94)
53	Chaloupka & Fong	2012	United States	Tobacco taxes as a tobacco control strategy(95)
54	Powell et al.	2012	United States	Assessing the potential effectiveness of food and beverage taxes and subsidies for improving public health: a systematic review of prices, demand and body weight outcomes(96)
55	Tiffin & Arnoult	2011	England	The public health impacts of a fat tax(97)
56	Ekins et al.	2011	England	A major environmental tax reform for the UK: results for the economy, employment and the environment(98)
57	Miguel & Manzano	2011	Spania	Gradual green tax reforms(99)
58	Dissou & Eyland	2011	Canada	Carbon control policies, competitiveness, and border tax adjustments(100)
59	Härkänen et al.	2011	Finland	The welfare effects of health-based food tax policy(101)
60	Uadiale et al.	2010	Nigeria	An empirical study of the relationship between culture and personal income tax evasion in Nigeria(102)
61	Scasny et al.	2009	Czech Republic	Analyzing macroeconomic effects of environmental taxation in the Czech Republic with the econometric E3ME Model(103)
62	DeCicca & McLeod	2008	United States	Cigarette taxes and older adult smoking: evidence from recent large tax increases(104)
63	Glomma & Sepulveda	2008	United States	Green taxes and double dividends in a dynamic economy(105)
64	Mayeres & Van Regemorter	2008	EU countries	Modelling the health related benefits of environmental policies and their feedback effects: a CGE analysis for the EU countries with GEM-E3(106)
65	Keen & Lockwood	2007	panel of 143 countries	The Value-Added Tax: Its causes and consequences(107)
66	Mytton et al.	2007	England	Could targeted food taxes improve health? (108)
67	O'Donoghue & Rabin	2006	United States	Optimal sin taxes(109)
68	Van Baal et al.	2006	Netherlands	Increasing tobacco taxes: a cheap tool to increase public health(110)
69	Gangadharan & Valenzuela	2001	51 countries	Interrelationships between income, health and the environment: extending the Environmental Kusnets curve hypothesis(111)
70	Schneider & Enste	2000	76 countries	Shadow economies around the world: size, causes, and consequences(112)

Table S3: Articles on types of health taxes (Persian)

No	Study title	Type and application of taxes
1	Stimating the Optimal Taxation Rate on Consumer Goods Harmful to Health by Microstimulating in Iran(1)	The optimal rate on harmful goods should be provided t, while having an efficient impact on household consumption patterns, certain tax revenue is obtained in exchange for a minimum reduction in welfare for households.
2	Feasibility Study of Implementing Green Tax System in Iran(2)	Given the importance of green taxes, governments, organizations, and companies should include them in their environmental policy portfolios so that they can offset their pollution costs with environmental activities or pay more taxes.
3	Evaluating the potential effect of tax policies on the consumption of soft drinks(3)	Applying a higher-than-standard rate in the new VAT law could decrease soft drink consumption.
4	Examining the role of value added tax on the development of social justice(5)	Value-added tax methods based on the development of social justice can increase the level of social welfare and the achievement of equality and social facilities by all classes of society, provide all members of society with them, and lead society and the country towards maximum development in all its aspects, including economic, political, cultural, and social development.
5	Quality and quantity of price elasticity of cigarette in Iran(6)	Economic measures, such as taxes that increase the price of cigarettes, are a useful policy tool to limit the initiation and intensity of smoking in Iran.
6	Rate structure, justice and exemption in the value added tax system(7)	Implementing value-added tax at different rates increases the general level of prices, controls consumption, increases government revenues, reduces the prices of production factors, and reduces household income and expenses.
7	Investigating the Effect of the Green Tax on Iran's Health Sector: A General Equilibrium Approach(12)	Imposing green taxes on non-environmentally friendly products and services is recommended as an efficient way to improve health indicators.
8	Modeling the effects of green tax on health spending using a computable general equilibrium model(10)	By increasing green tax rates, health system costs resulting from reducing air pollution will be significantly reduced.
9	Analyzing the Impact of Green Tax on Emission of Pollution and Health Index in Iran: A Simultaneous Equations Model(14)	Imposing green taxes will reduce pollutant emissions and, as a result, increase the health index.
10	Value added tax in goods and services harmful to health(16)	To reduce the consumption of goods that are harmful to health, improve the health level of the community, and replace the consumption of these goods with healthy goods, the cost of consuming these goods can be increased by using strategies such as setting a specific tax rate (on sales or value added) and duties on the aforementioned goods.
11	explanation of Interaction between Iranian Physicians and Government in the Field of Tax: A Qualitative Study(19)	The government should provide doctors with various welfare facilities to encourage them to pay the appropriate taxes.
12	Application of the endogenous growth model to calculate the optimal value added tax rate with an emphasis on harmful and waste products(20)	With the increase in social sensitivity to harmful and wasteful goods, the appropriate rate of value-added tax should be increased to provide appropriate conditions for social welfare.
13	The effect of pollution tax on houshould's demand for environment polluting goods(23)	The significant impact of taxes on reducing the consumption of environmentally polluting goods makes the use of tax instruments as an effective solution to reduce pollution important, and policymakers should, therefore, consider it.
14	Two different rates on value added tax(27)	Two different tax rates are determined for luxury goods and other goods: The tax rate on food is 8%, and the tax rate on luxury goods is about 26%
15	Prediction of value added tax due to tobacco use in Iran using neural network method(28)	Since one of the policymakers' concerns is accurate predictions of tax revenues, it is better to use the neural network method to predict the tobacco VAT.
16	Green taxes and environmental standards of imports, appropriate tools of sustainable development in Iran's transition economy(29)	Reforming environmental laws and focusing on sustainable development using strict environmental laws are important in reducing pollutant pollution in developing countries
17	Green tax forgetting factor in Iranian industrial planning(30)	In terms of policymaking, it is necessary to provide measures to prevent environmental degradation by applying green taxes.
18	Investigating the effect of VAT on income distribution in selected countries(39)	The government should establish a VAT on goods in public consumption in the country in proportion to their price and income elasticity.
19	The Survey of Welfare and Environmental Effects of Green Tax and Decline Subsidy on Fueles in Iran by Using a Computable General Equilibrium Model(40)	Using green taxes within the framework of the country's tax system can be significantly effective in reducing air pollution.

Table S4: Articles on types of health taxes (English)

No	Study Title	Tax
1	Sin taxes and their effect on consumption, revenue generation and health improvement: a systematic literature review in Latin America(44)	Taxes on unhealthy foods and beverages effectively reduce the consumption of potentially harmful products, improve public health, and generate additional revenue
2	Taxes to Unhealthy Food and Beverages and Oral Health in Mexico: An Observational Study(45)	Taxes on unhealthy foods and beverages have a positive impact on oral health.
3	Estimating the impact of tax policy interventions on the projected number and prevalence of adults with type 2 diabetes in Germany between 2020 and 2040(46)	Taxes on sugar-sweetened beverages, tobacco products, and red meat would reduce the projected incidence and prevalence of type 2 diabetes in adults by 50 percent by 2040.
4	Food and beverage industry interference in science and policy: efforts to block soda tax implementation in Mexico and prevent international diffusion(48)	The food and beverage industry trade organizations and interest groups are lobbying some experts and researchers to produce research showing that taxes on their products fail to improve public health and harm the economy.
5	Employment impacts of the San Francisco sugar-sweetened beverage tax 2 years after implementation(49)	Two years after the tax on unhealthy foods was introduced, there is no evidence that this type of taxation has hurt net employment, private sector employment, or employment in specific industries related to unhealthy foods.
6	Do taxes on unhealthy foods and beverages influence food purchases(50)	Taxes on unhealthy foods and beverages are promising strategies for improving public diets.
7	How do consumers respond to "sin taxes"? New evidence from a tax on sugary drinks(51)	Taxation effectively reduces the purchase of taxed unhealthy drinks and slightly increases the purchase of tax-free drinks. This results in a 2.2% reduction in sugar purchases.
8	Do environmental taxes reduce air pollution? Evidence from fossil-fuel power plants in China(52)	Environmental taxes have a positive impact on reducing pollutant emissions compared to many other pollution control policies. Furthermore, there is an inverse relationship between pollutant emissions and tax rates.
9	Optimal sin taxes in the presence of income taxes and health care(53)	Taxing harmful and unhealthy products has a double benefit: It improves not only the well-being and health of society but also the economic performance of countries.
10	The potential global gains in health and revenue from increased taxation of tobacco, alcohol and sugar- sweetened beverages: a modelling analysis(55)	Increasing indirect taxes on tobacco, alcohol, and sugar-sweetened beverages significantly reduces premature mortality and increases government revenues (thereby increasing public health budgets).
11	Do environmental taxes and environmental stringency policies reduce CO2 emissions? Evidence from 7 emerging economies(56)	Stringent environmental policies and imposing environmental taxes are two practical tools for combating negative environmental impacts.
12	Impact of cigarette tax increase on health and financing outcomes in four Indian states(58)	Tobacco tax policies play an important role in achieving public health outcomes in low- and middle-income countries and contribute to community poverty reduction.
13	The health and financial impacts of a sugary drink tax across different income groups in Canada(60)	A 20% tax on sugary drinks reduces consumption by an average of about 15%. An estimated 20% tax on sugary drinks would prevent about 690,000 lifetime DALYs among adults annually.
14	Comparing selected countries using sin tax policy in sustainable health financing: Implications for developing countries(61)	Taxing harmful products such as tobacco and alcohol is a key policy for sustainable public health financing in all countries.
15	Impacts of excise taxation on non-alcoholic beverage consumption in Vietnam(62)	A 10% tax on alcohol would reduce consumption by 11.4%.
16	A call to action: advocating for the integration of oral health promotion and public health via sugar sweetened beverage taxation(64)	A tax on sugar-sweetened beverages would reduce sugar consumption, potentially preventing dental caries.
17	Understanding heterogeneity in price changes and firm responses to a national unhealthy food tax in Mexico(66)	Given the high prevalence of overweight and obesity and the need to control them in Mexico, an 8% VAT on unhealthy foods was approved and implemented.
18	Tax design in the alcohol market(67)	Imposing different tax rates on different forms of alcohol can lead to a significant increase in social welfare.
19	Regressive sin taxes, with an Application to the Optimal soda tax(68)	Imposing a tax on a product with negative effects increases social welfare by reducing consumption to its optimal level, so the marginal social cost is equal to the marginal benefit.
20	Sin tax as a public health tool-strengths and weaknesses of this economic solution(69)	If implemented properly, imposing a tax on harmful products can help change consumer behavior and have a positive impact on society's health.
21	The Use of Excise Taxes to Reduce Tobacco, Alcohol, and Sugary Beverage Consumption(71)	Excise taxes are the most effective tax on tobacco, alcohol, and unhealthy beverages.<br>Taxes based on the ethanol content of alcoholic beverages and the sugar content of sugar-sweetened beverages will reformulate the ingredients of these products, thereby increasing their positive impact on public health.

22	Potential health impacts of increasing the cigarette tax in Viet Nam(72)	Increasing taxes on tobacco, including cigarettes, can significantly reduce the adverse effects of their consumption and prevent the waste of 57 billion USD in financial resources in Vietnam.
23	Optimal carbon taxes for China and implications for power generation, welfare and the environment(73)	A carbon tax will reduce carbon production in various sectors, including electricity and environmental pollution, by about 62.5% and increase social welfare
24	The Association Between State Value-Added Taxes and Tobacco Use in India—Evidence From GATS and TCP India Survey(74)	High VAT rates on cigarette consumption are significantly associated with decreased cigarette consumption.
25	Appetite for health-related food taxes: New Zealand stakeholder views(75)	Taxes on sugar-sweetened beverages and subsidies can be associated with children's health, including oral health.
26	Health-motivated taxes on red and processed meat: A modelling study on optimal tax levels and associated health impacts(76)	Taxes on red and processed meats are associated with reduced consumption of these foods, the number of deaths related to them, and health care costs, with this reduction being most pronounced in high—and middle-income countries.
27	Should we tax unhealthy food and drink? (77)	Taxes on unhealthy food and beverages can potentially improve public health and reduce the incidence of related diseases.
28	The impact on productivity of a hypothetical tax on sugar-sweetened beverages(81)	A 20% tax on sugar-sweetened beverages would reduce obesity rates in the general population by 1.96%. In addition, the tax would save 35,000 life years and reduce healthcare costs by $425 million.<br>In addition, a 10% tax on sugar-sweetened beverages (SBB) would reduce consumption by 6% per year.
29	Policy lessons from health taxes: a systematic review of empirical studies(83)	Increasing tax rates on SBB consumption is likely to have a positive impact on health behaviors and outcomes. This means that taxes on these products reduce demand while increasing fiscal revenues.
30	Changes in prices, sales, consumer spending, and beverage consumption one year after a tax on sugar-sweetened beverages in Berkeley, California, US: A before-and-after study(85)	A year after the first tax on sugar-sweetened beverages (SSDs) was implemented, prices of these beverages increased in many cases, sales fell, and sales of tax-free beverages (especially water) and other similar beverages increased in the US state of California.
31	Modelling the potential impact of a sugar-sweetened beverage tax on stroke mortality, costs and health-adjusted life years in South Africa(87)	Taxing sugar-sweetened beverages, especially a VAT tax, could prevent an estimated 72,000 deaths and 550,000 years of healthy life lost due to stroke. Such policies could also save $5 billion in health care costs.
32	The impact of the French soda tax on prices, purchases and tastes: an ex post evaluation(88)	Taxing highly sugary beverages would increase prices by 20 percent, affecting calorie intake, weight gain, and non-communicable diseases.
33	Econometric and comparative risk assessment scenario modelling of the proposed UK sugary drink tax on health(90)	Implementing the SBB tax in the beverage industry has significant positive effects on the population's health.
34	Tobacco taxes as a tobacco control strategy(95)	Significantly increasing the tax rate reduces tobacco consumption, especially among young people and people with low incomes. This increase is a very effective strategy for tobacco control and can lead to improvements in public health.
35	Assessing the potential effectiveness of food and beverage taxes and subsidies for improving public health: a systematic review of prices, demand and body weight outcomes(96)	Taxing fast food is associated with less weight loss, especially among adolescents.
36	A major environmental tax reform for the UK: results for the economy, employment and the environment(98)	Taxing goods reduces the consumption of polluting goods by increasing the price of goods.
37	The welfare effects of health-based food tax policy(101)	Taxing sugar significantly reduces the incidence of type 2 diabetes and coronary heart disease in the population. The health effects seem to be more pronounced for low-income people, so implementing health-oriented food tax reforms may reduce health inequalities in the population.
38	Analyzing macroeconomic effects of environmental taxation in the Czech Republic with the econometric E3ME Model(103)	Implementing environmental policies such as taxes increases the price of fossil fuels and reduces the pollution they generate.
39	Cigarette taxes and older adult smoking: evidence from recent large tax increases(104)	Increasing the tax rate on tobacco reduces smoking among older people, especially those with less education and in low-income families.
40	Green taxes and double dividends in a dynamic economy(105)	Increasing the gasoline tax and reducing the capital tax have two types of welfare effects on society: increasing the consumption of goods (efficiency benefit) and improving the quality and improvement of the environment (green benefit).
41	The Value-Added Tax: Its Causes and Consequences(107)	The imposition of a value-added tax has increased efficiency in many of the countries that have implemented it.
42	Optimal sin taxes(109)	Imposing taxes on harmful goods and returning the revenue to consumers improves social welfare and the community's health.
43	Interrelationships between income, health and the environment: extending the Environmental Kusnets curve hypothesis(111)	Improving environmental indicators, such as life expectancy, infant mortality, and child mortality, is related to the health status of the community.

Table S5: SWOT analysis of health taxes (Persian articles)

No	Study Title	Peresent Situation
1	stimating the Optimal Taxation Rate on Consumer Goods Harmful to Health by Microstimulating in Iran(1)	The oil and fat groups and the tobacco group, with price elasticities of -0.381 and -0.478, respectively, have the lowest elasticity among the harmful commodity groups, and therefore, households' sensitivity to price changes in these two groups is low. The income elasticities of the oil and fat and tobacco groups, 0.45 and 0.37, respectively, show that these goods are considered essential compared to other groups. Tax policies (price) and household education policies can affect the consumption levels of these two groups.
2	Evaluating the potential effect of tax policies on the consumption of soft drinks(3)	Examining the effects of welfare using the compensatory change criterion shows that increasing the tax rate does not significantly reduce consumer welfare. However, households in the upper-income deciles suffer the most significant welfare losses. Therefore, the correct application of tax policies in the VAT law can have a positive and significant effect on controlling the consumption of soft drinks.
3	Quality and quantity of price elasticity of cigarettes in Iran(6)	Non-Iranian cigarette brands have the largest share of expenditure in Iranian families and the highest consumption traction among cigarette types. The effectiveness of tax policies depends on different brands of foreign cigarettes.
4	Investigating of the tax income and oil revenues on health expenditures in Iran(9)	Tax and oil revenues positively and significantly impact health spending in Iran.
5	Simulation of Green Tax Effects on Health Indicators and Welfare in Iran: General Equilibrium Model of Economy, Energy and Environment (GEM-E3)(13)	The financial assessment of the impact of pollutants on health indicators, including deaths and illnesses and other related issues caused by air pollution, was estimated at 62, 4.26, and 11.6 percent, respectively. Redistribution of revenue from green taxes increases society's welfare and the scenario of helping pollution-reducing technologies.
6	Investigating the Effect of the Green Tax on Iran’s Health Sector: A General Equilibrium Approach(12)	Considering that Iran has one of the highest per capita energy consumption rates, pollution threatens the country. The imposition of a green tax will reduce all types of environmental pollution. Also, 62% of health costs are spent on mortality, 26.4% on disability, and 11.6% on non-health outcomes.
7	Modeling the effects of green tax on health spending using a computable general equilibrium model(10)	Currently, the government should spend part of the revenue from the green tax on pollution-reducing technologies, especially in polluting industries, and the other part on helping the health sector.
8	Analyzing the Impact of Green Tax on Emission of Pollution and Health Index in Iran: A Simultaneous Equations Model(14)	The government should reduce carbon dioxide emissions in the industrial production process by imposing a tax on carbon dioxide emissions and implementing environmental programs, thereby increasing society's health index.
9	Review of green tax and double benefit in Iran's economy(15)	By imposing taxes on polluting sectors, in all scenarios, considering the positive effect of reducing pollution, changes in the welfare of the society are positive, and its amount increases with increasing tax rates, but production has decreased in all scenarios; as a result, the double benefit is not achieved in Iran by imposing a green tax, and the highest welfare growth rate for a tax rate of 4 percent is considered an appropriate rate.
10	Value added tax in goods and services harmful to health(16)	Increasing the price of goods while changing households' consumption patterns and replacing the aforementioned goods with healthy goods in their consumption baskets can lead to a change in production lines or replacing unhealthy raw materials with less harmful or non-harmful goods.
11	Effects of Green Tax on Economic Growth and Welfare in Economy of Iran: a Dynamic Stochastic General Equilibrium Approach (DSGE)(18)	Establishing a green tax leads to a sustainable and slight reduction in pollution. Considering the simultaneous results of the effects of green taxes on economic growth, social welfare, and pollution, it is clear that if the government's approach is to pay attention to increasing environmental quality, reducing pollution, sustainable development, and increasing social welfare, it must accept a decrease in economic production.
12	Explanation of Interaction between Iranian Physicians and Government in the Field of Tax: A Qualitative Study(19)	The Tax Administration does not have sufficient access to physicians' income information to determine their taxes.
13	Application of the endogenous growth model to calculate the optimal value added tax rate with an emphasis on harmful and waste products(20)	Higher economic growth will increase the appropriate tax rate. Considering the variables and parameters of the Iranian economy, the appropriate value-added tax rate for the Iranian economy with harmful goods and waste is 21 percent, and without considering harmful goods and waste, it is 19 percent.
14	Identify barriers and drivers of establishment of VAT(21)	Three groups of obstacles have been identified as cultural, administrative, and structural obstacles to establishing the value-added tax system.
15	The Status of Television Advertisements of Health-threatening Products(22)	Given the high level of advertising of goods harmful to health while continuously monitoring advertising, appropriate regulatory and legal solutions should be used in this field, and new deterrent laws should be developed and implemented.
16	The effect of pollution tax on household’s demand for invironment polluting goods(23)	The tax elasticity of demand for environmentally polluting goods, including vehicle fuel, electricity, and water, shows that imposing taxes reduces the consumption of these goods and thereby reduces pollution emissions. In addition, the effect of a tax on vehicle fuel on reducing its consumption is stronger than the effect of a tax on the consumption of other goods.
17	dentify barriers to optimal implementation of the VAT system in Iran(24)	The complexity of the tax administration, the lack of adequate and appropriate information, the weakness of appropriate software, and the lack of adequate training of tax officials affect the proper implementation of the value-added tax system.

18	Empirical Analysis of the relationship between air pollution and public health expenditures - A dynamic panel data approach(25)	Air pollution has a positive and significant effect on increasing public health costs.
19	Two different rates on value added tax(27)	Tax rate reform is one of our society's necessities, but changing tax policies should be done gradually with institutional and political reform. In implementing this tax plan, a connection between tax policy and the behavior of firms and households must be established, and this connection is very important for policymaking.
20	Green tax forgetting factor in Iranian industrial planning(30)	The profitability of polluting industrial products seems significantly higher than that of clean industries.
21	Estimating the optimal level of pollution tax in Iranian economy(32)	Taxing air pollution will increase social welfare (by reducing the emission of environmental pollutants) and decrease social welfare (reducing production and consequently reducing consumption). The result of these two effects can increase or decrease social welfare in general.
22	Investigate the interactions between economic growth and environmental destruction on health(36)	The relationship between countries' incomes and environmental pollution is positive, and the effect of income on the health index is positive, while the effect of pollution on the health index is negative.
23	Estimating the elasticity of demand for goods and services subject to consumption and sales tax in Iran(41)]	In terms of income sensitivity, tobacco, personal services, cosmetics and decorative items, transportation, communications, clothing, and footwear are considered luxury goods, and beverages, water and fuel and lighting, household appliances and furniture, tea and coffee, and cocoa are considered essential goods.<br>In terms of price sensitivity, all groups except transportation, water, fuel, lighting, and clothing and footwear are inelastic.

Table S6: SWOT analysis of health taxes (English articles)

No	Study title	Peresent Status
**1**	Linking health and finance ministries to improve taxes on unhealthy products(43)	New approaches to supporting health taxation include prioritizing health system financing due to the growing burden of non-communicable diseases and addressing the health and economic harms caused by pandemics such as the coronavirus. As a result, high-level efforts to achieve progress in health taxation are gaining momentum and represent an important step forward in harnessing the combined expertise of health and finance policymakers.
2	Estimating the impact of tax policy interventions on the projected number and prevalence of adults with type 2 diabetes in Germany between 2020 and 2040(46)	Although increasing taxes on unhealthy products is currently practical as a stand-alone measure, it may not be sufficient to reduce diabetes prevalence in the future.
3	South Africa's Health Promotion Levy: Excise tax findings and equity potential(47)	Despite reducing taxes on sugar-sweetened beverages (SSDs) from 20 to 10 percent, purchases of unhealthy SSDs and sugar consumption have decreased. There have also been more significant reductions in SSD purchases among lower socioeconomic groups and in subpopulations with higher SSD consumption.<br>These subpopulations bear a more significant burden of obesity and related diseases, suggesting that the policy improves health equity.
4	Food and beverage industry interference in science and policy: efforts to block soda tax implementation in Mexico and prevent international diffusion(48)	Resistance from industries such as the beverage industry could persist after new policies become law, as vested interests seek to oppose legislation or prevent the implementation of tax policies.
5	Do taxes on unhealthy foods and beverages influence food purchases(50)	Evaluations of taxes on unhealthy foods and beverages suggest that they reduce the purchase of unhealthy products and nutrients. However, the potential for substitution with other unhealthy foods should be considered in tax design.
6	How do consumers respond to "sin taxes"? New evidence from a tax on sugary drinks(51)	Although taxes on harmful products change consumer behavior, a combination of policies is needed to address chronic disease.
7	Do environmental taxes reduce air pollution? Evidence from fossil-fuel power plants in China(52)	Policymakers could consider raising tax rates in low-tax areas and strengthening tax exemptions to encourage companies to reduce emissions.
8	Health Taxes on Tobacco, Alcohol, Food and Drinks in Low- and Middle-Income Countries: A Scoping Review of Policy Content, Actors, Process and Context(54)	Given the prevalence of non-communicable diseases in LMICs, identifying and carefully exploring the economic and political factors influencing the design and implementation of fiscal measures, especially taxes, is important.Further investigation is required to determine whether the mechanisms used to impose tobacco taxes are applicable to targeting measures on foods, alcohol, and other beverages.
9	The effects of environmental tax reform on urban air pollution: A quasi-natural experiment based on the Environmental Protection Tax Law(57)	Air pollution significantly affects sustainable development and public health. There are four important issues in this regard.<br>1. Environmental tax reform can significantly reduce urban air pollution.<br>2. Green technology innovation and upgraded industrial structure are a crucial transmission mechanism for environmental tax reform to improve air quality.<br>3. Environmental tax reform significantly prevents urban air pollution in various cities, huge cities.

		4. Furthermore, environmental tax reform promotes improvements in local air quality and reduces air pollution in nearby cities.
10	Industry strategies in the parliamentary process of adopting a sugar-sweetened beverage tax in South Africa: a systematic mapping(59)	The industry uses several tactics to oppose a tax on sugar-sweetened beverages. First, the industry emphasizes its economic importance and the potential job losses and other economic harms that could result from the tax.<br>Second, the industry argues for self-regulation and voluntary action as a policy alternative.<br>Third, the industry misrepresents or denies evidence to undermine the tax's effectiveness.
11	Comparing selected countries using sin tax policy in sustainable health financing: Implications for developing countries(61)	The Iranian health system does not have significant legal and political gaps, but there are limitations in implementing tax policies.<br>The primary sources of financing in the selected countries included health promotion funds with different names and objectives that tax harmful goods, tobacco, and alcohol.<br>Weak implementation and monitoring of tax laws were the main reasons for the lack of sustainable financing.
12	Impacts of excise taxation on non-alcoholic beverage consumption in Vietnam(62)	With increasing taxes, consumers are substituting alternative goods, such as milk, tea, and alcoholic beverages, for non-alcoholic beverages.<br>Tax increases also decrease consumer welfare due to the increase in the price of sugary drinks.
13	The effect of implicit and explicit taxes on the purchasing of 'high-in-calorie' products: A randomized controlled trial(63)	Although taxes on less healthy food and beverage products are effective, it is unclear whether the observed reduction in taxable products purchased results from price increases due to taxes or accompanying messages. Policymakers should be aware that changes in price and purchasing patterns may not improve diet quality.
14	A call to action: advocating for the integration of oral health promotion and public health via sugar sweetened beverage taxation(64)	SBB taxes can be an effective way to integrate oral health with public health promotion. However, the revenues from the SBB tax should be invested in promoting oral health.
15	Equity as a consideration in evaluations of health taxes: a systematic review(65)	Despite the recommendations, the assessment of health-related taxes does not typically consider distributive justice in order to be beneficial.
16	Sin tax as a public health tool-strengths and weaknesses of this economic solution(69)	Taxing harmful goods is undoubtedly an effective and practical tool, but it has flaws. Its primary drawbacks are its regressive nature and potential negative social perception.
17	Public support for health taxes and media regulation of harmful products in South Korea(70)	The South Korean public strongly supports the introduction of health-related taxes and media regulations on advertising unhealthy products.
18	The use of excise taxes to reduce Tobacco, Alcohol, and Sugary Beverage Consumption(71)	Tobacco taxes, given the relative risks of these products, could help to shift some smokers to less harmful nicotine. The potential for these taxes to significantly reduce consumption and save lives remains high.
19	Potential health impacts of increasing the cigarette tax in Viet Nam(72)	Strengths Increasing tobacco taxes, especially on cigarettes, for young people could have a significant impact on health and reduce tobacco use, leading to significant financial savings across society.
20	Health-motivated taxes on red and processed meat: A modelling study on optimal tax levels and associated health impacts(76)	Including social health costs in red and processed meat pricing could lead to significant health and environmental benefits, particularly in high- and middle-income countries
21	Should we tax unhealthy food and drink? (77)	Health-related food tax should be carefully designed and evaluated to minimize unintended consequences and maximize public health. Taxes alone will not reduce the burden of diet-related diseases. However, they can make an important contribution to changing the behavior of the manufacturing and consumer industries in the right direction.
22	State-of-the-art for food taxes to promote public health (78)	The diversity of experiences with existing tax schemes provides valuable insights into the future use of financial instruments to promote healthy eating in terms of designing effective and efficient tax or subsidy instruments and in terms of policy and implementation.
23	Sugar-Sweetened Beverage Taxes: Industry Response and Tactics(79)	With increasing public awareness of the link between sugar and obesity, the sugar industry is increasingly seeking to remove the blame from its products. It is actively lobbying to oppose the passage of a tax on sugary drinks.
24	Taxation of sugar sweetened beverages and unhealthy foods: a qualitative study of key opinion leaders' views(80)	Potential obstacles to a tax on sugary drinks include opposition from various sectors, technical and bureaucratic obstacles to implementing such a tax, difficulties in determining the tax on these products, and opposition to allocating tax revenues to public health. Therefore, introducing a health-related tax should be part of a multi-pronged strategy. Tax revenues should also be appropriately allocated, particularly for public health promotion, obesity treatment and prevention, and education on healthy eating.
25	Economics and environmental implications of fuel efficiency improvement in Malaysia: A computable general equilibrium approach(82)	Implementing fuel tax policies and improving efficiency are adequate and appropriate measures to reduce environmental pollution emissions.<br>If the primary policy objective of allocating taxes to health is to reduce the consumption of unhealthy products, this would support the implementation of taxes that increase the price of products by 20% or more.
26	Policy lessons from health taxes: a systematic review of empirical studies(83)	If the primary policy objective of allocating taxes to health is to reduce the consumption of unhealthy products, this would support the implementation of taxes that increase the price of products by 20% or

		more.Therefore, policy actors should be transparent about reducing the production of unhealthy products by allocating health-related taxes and setting taxes accordingly.
27	Taxes and Subsidies for Improving Diet and Population Health in Australia: A Cost Effectiveness Modelling Study(84)	Allocating health-related taxes can be combined to achieve system improvements and cost savings in the health sector. Therefore, more attention should be paid to the prominent role of tax packages in improving public health.
28	Changes in prices, sales, consumer spending, and beverage consumption one year after a tax on sugar-sweetened beverages in Berkeley, California, US: A before-and-after study(85)	The significant reduction in the sale of sugar-sweetened beverages and the revenue used for prevention promises a positive impact on tax policy.
29	Framing the tax and health nexus: a neglected aspect of public health concern(86)	Allocating health-related taxes can help governments be more responsive to citizens' needs and generate revenue for health care and other public services. Progressive taxation can help redistribute wealth and income and reduce social and health inequalities.<br>Repricing harmful products (such as tobacco, alcohol, and junk food) helps reduce their consumption and ultimately provides a way for some harmful industries to be regulated appropriately.
30	Modelling the potential impact of a sugar-sweetened beverage tax on stroke mortality, costs and health-adjusted life years in South Africa(87)	A tax on sugary drinks, part of a multi-pronged approach, contributes to the health system's goal of reducing premature mortality (among people under 60).
31	The impact of the French soda tax on prices, purchases and tastes: an ex post evaluation(88)	Despite the growth and development of tax instruments worldwide, they are still incomplete and, in some cases, even conflict with each other.
32	Hard to Avoid but Difficult to Sustain: Scotland's Innovative Health Tax on Large Retailers Selling Tobacco and Alcohol(89)	Health-related taxes raise revenue for health spending and help achieve health goals (behavior change), but there may be trade-offs between these goals in the design of relevant policies. Allocating taxes as a health system support measure may increase public support in the short term. However, this may not be sustained if such a framework is not sufficiently introduced and publicized.
33	Econometric and comparative risk assessment scenario modelling of the proposed UK sugary drink tax on health(90)	Data from the SBB tax policy exercise can be used to help and advise the UK government on maximizing the impact of taxes on health.
34	Financing universal health coverage effects of alternative tax structures on public health systems: cross-national modelling in 89 low-income and middle-income countries(91)	Increasing tax revenues is essential to achieving universal health coverage, particularly in countries with low tax bases. Pro-poor tax policies may accelerate progress toward achieving key health goals.
35	Health spending, illicit financial flows and tax incentives in Malawi(92)	There is a significant gap between government spending on health and the costs of delivering health services and packages, and this gap could be closed through tax incentives such as reforming the law, improving transparency, limiting tax evasion, and helping to reduce the incidence of disease in society.
36	Tax expenses and their impacts on performance in health and education(93)	Tax spending in Latin America is rising faster than in OECD countries. Countries that use higher tax allocations may have poorer health and social indicators.
37	Taxes on Sugar-Sweetened Beverages: Results from a 2011 National Public Opinion Survey(94)	Without strong public support for health-related taxes, it is challenging to implement tax policies.
38	Tobacco taxes as a tobacco control strategy(95)	When revenues from tobacco tax increases are used to support tobacco control, health promotion, or other health-related activities and programs, significant positive health impacts are observed. Generally, arguments that tax increases will have harmful economic effects are false or exaggerated.
39	Assessing the potential effectiveness of food and beverage taxes and subsidies for improving public health: a systematic review of prices, demand and body weight outcomes(96)	Pricing instruments can be potential policy tools to address health-related risk factors.
40	The public health impacts of a fat tax(97)	Tax-based interventions should be considered among a range of policy interventions, including policies aimed at improving highly unhealthy diets.
41	Gradual green tax reforms(99)	Green tax reforms are important for protecting the environment and making the tax system more efficient.
42	Carbon control policies, competitiveness, and border tax adjustments(100)	Taxes on polluting industries have been shown to improve conditions in some countries. However, conditions have not improved in other countries, and the situation varies from country to country.
43	Modelling the health related benefits of environmental policies and their feedback effects: a CGE analysis for the EU countries with GEM-E3(106)	Explicit modeling of the health effects of air pollution would provide a better assessment of the impact of environmental policies on manufacturing industries and job creation in these industries.
44	Could targeted food taxes improve health?(108)	Taxing only the primary sources of saturated fat in the diet would not reduce cardiovascular disease, as increased salt intake would offset the reduction in saturated fat. In addition, taxing unhealthy foods could prevent around 2,300 deaths per year.

		Taxing a wider range of foods could prevent up to 3,200 deaths from cardiovascular disease in the UK per year. If the cross-elasticities of demand are ignored, food taxes could have unpredictable health effects.
45	Optimal sin taxes(109)	Taxing harmful goods would reduce consumers' excessive consumption while redistributing income among all consumers.
46	Increasing tobacco taxes: a cheap tool to increase public health(110)	Even if tax revenues are not allocated to the health system, increasing taxes effectively improves public health.

References:

1. Salem A A, Gholami E. Estimating the Optimal Taxation Rate on Consumer Goods Harmful to Health by Microstimulating in Iran. Quarterly Journal of Economic Research and Policies. 2022; 30(101): 85-119 [Persian]
2. Rasmi, Abedi, Panahi, mousavi, yeganeh. Feasibility Study of Implementing Green Tax System in Iran. J Sus Dev & Env. 2022;3(1):45-61 [Persian]
3. Farzin Mehr HR, Lashkary M, Bafandeh Imandoust S. Evaluating the potential effect of tax policies on the consumption of soft drinks. Journal of Tax Research. 2022;29(52):53-78 [Persian]
4. Sameti M, Izadi A, Fathi S. Determining factors affecting tax evasion using meta-analysis method. Stable Economy Journal. 2021; 2(2): 1-22, Doi: org/10.22111/sedj.2021.38231.1113 [Persian]
5. Choobineh B, Samadi A H, Hadian E, Dehghan Shabani Z. The Effect of Tax Evasion on Monetary Rule under Fiscal Dominance and Liquidity Constraint: The Case of Iran. Quarterly Journal of Economic Research and Policies. 2021; 29(98): 289-326 [Persian]
6. Homaie Rad E, Habibullah Pulok M, Rezaei S, Reihanian A. Quality and quantity of price elasticity of cigarette in Iran. Health planning and management. 2020: 36(1): 60-70, Doi: org/10.1002/hpm.3062 [Persian]

7. Aghaei M, Nahid A. Rate structure, justice and exemption in the value added tax system. Majlis and Research. 2004; 11(45): 147-70 [Persian]
8. Sanaeepour H. Identifying and Prioritizing the Factors Influencing Tax Evasion in Small & Medium Enterprises (SMEs) from the Perspective of Iranian National Tax Administration Staff: A Mixed Method Study. Journal of Tax Research. 2020; 28(47): 7-30 [Persian]
9. Nadri S, Khodabakhshi A. Investigating of the tax income and oil revenues on health expenditures in Iran. Quarterly Journal of Applied Economics Studies Iran (AESI). 2019; 8(31): 255-75, Doi: org/10.22084/aes.2019.17608.2748 [Persian]
10. Torki Harchegani M.A, Dahmardeh N. Modeling the effects of green tax on health spending using a computable general equilibrium model. Journal of Economic Modeling. 2018; 12(3): 79-97 [Persian]
11. Ezzati Gharibdosti S, Qaem Panah S. The legal loopholes of tax evasion in Iran's financial laws. Political and international studies. 2019; (40): 243-60 [Persian]
12. Torki Harchegani M.A, Dahmardeh N. Investigating the Effect of the Green Tax on Iran's Health Sector: A General Equilibrium Approach. Iranian Journal of Economic Studies. 2017; 6(2): 251-70, Doi: org/10.22099/ijes.2018.28305.1430 [Persian]
13. Torki Harachgani M. Simulation of Green Tax Effects on Health Indicators and Welfare in Iran: General Equilibrium Model of Economy, Energy and Environment (GEM-E3). Journal of Tax Research. 2023;31(59):28-48 [Persian]
14. Izadkhasti H, Arabmazar AK, Khoshnamvand. Analyzing the Impact of Green Tax on Emission of Pollution and Health Index in Iran: A Simultaneous Equations Model. Quarterly Journal of Economics and Modeling. 2017; 8(29): 89-117 [Persian]
15. Jabari A, Firouzeh GH. 2016, Review of green tax and double benefit in Iran's economy, World National Conference on Scientific Research in Management, Accounting, Law and Social Sciences, Shiraz, Islamic Azad University, Zarghan Branch - Shoshtar University of Applied Sciences [Persian]
16. Afshari F, Javadi Nasab H, Valizadeh B. Value added tax in goods and services harmful to health. National conference on value added tax: opportunities and challenges. 2017 [Persian]
17. Keshtkaran A, Heidari AR, Keshtkaran V, Taft V, Almasi A. Satisfaction of outpatients referring to teaching hospitals clinics in Shiraz, 2009. Payesh (Health Monitor). 2012;11(4):459-65 [Persian]
18. Ahangari A, Farazmand H, Montazer Hojjat A H, Haft Lang R . Effects of Green Tax on Economic Growth and Welfare in Economy of Iran: a Dynamic Stochastic General Equilibrium Approach (DSGE). Journal of Quantitative Economis (Quartely Journal of Economics Review). 2018;15(1):27-61 [Persian]
19. Pakdaman M PA, Emamgholipour Sefiddashti S, Rahimi Foroushani A, Abdoli GH. . Explanation of Interaction between Iranian Physicians and Government in the Field of Tax: A Qualitative Study. 2016; 8(2): 1933-42. https://doi.org/10.19082/1933.

20. Jafari Samimi A KPS, Azami K. Application of the endogenous growth model to calculate the optimal value added tax rate with an emphasis on harmful and waste products. Economic Modeling Quarterly. 2015; 10(2): 65-114[Persian]
21. Mobini M MTGR, EbrahimZadeh A. Identify barriers and drivers of establishment of VAT. Journal of Development and Transformation Management. 2015; 24(1395): 23-32 [Persian]
22. Etemad K HAR, Lotfi M. The Status of Television Advertisements of Health-threatening Products. Journal of Health Research in Community. 2016; 2(3): 78-82[Persian]
23. Hosseinzadeh Kandsari Z MM. The effect of pollution tax on houshould's demand for environment-polluting goods. Journal of Environmental Science and Technology. 2018; 20(3): 105-15. https://doi.org/10.22034/jest.2018.13259.
24. Ziai Bigdeli M.T KRH. Identify barriers to optimal implementation of the VAT system in Iran. Iranian Journal of Public Administration Mission. 2014; 4(2): 1-15.
25. Fattahi M EA, Sadeghi H, Asgharpour H. Empirical Analysis of the relationship between air pollution and public health expenditures - A dynamic panel data approach. Quarterly Journal of Economic Modelling. 2016; 9(31); 43-60.
26. Amiri M. Behavioral economics and tax evasion. Economic Research Quarterly. 2017; 17(64): 95-130. https://doi.org/10.22054/joer.2017.7670 [Persian]
27. Seyed Nourani S.M, Mohammadi T, Amirshahi S. Two different rates on value added tax. Quarterly Journal of Economic Research and Policies. 2015; 23(73): 69-92. http://qjerp.ir/article-1-1112-fa.html [Persian]

28. Mousavi Jahromi Y, Gholami E. Prediction of value added tax due to tobacco use in Iran using neural network method. Economic Modeling Research Quarterly. 2015; 6(20): 55-72 [Persian]
29. Khosh Akhlagh R, Vaez Barzani M, Sadeghi Amrabadi B, Yarmohammadian N. Green taxes and environmental standards of imports, appropriate tools of sustainable development in Iran's transition economy. Agricultural Economics. 2014; 8(2): 175-95 [Persian]
30. FeizPour M.A, ShahMohamadi A, Asayesh F. Green tax forgetting factor in Iranian industrial planning. Journal of Environmental Studies. 2014; 40(2): 401-13, Doi: org/10.22059/jes.2014.51208 [Persian]
31. Rousta M, Heidarieh S A. Ranking causes of tax evasion by Analytic Hierarchy Process (AHP). Journal of Tax Research. 2014; 22(24): 157-73 [Persian]

32. Hadian E OAH. Estimating the optimal level of pollution tax in Iranian economy. Journal of Scientific and Research: Economic Growth and Development Research. 2013; 3(12): 57-74.
33. Samadi A.H TR. Tax Evasion in Iran: its Causes, Effects and Estimation. Journal of tax research. 2013; 21(19): 77-106.
34. Mahmood M, Mahmood N-m, Mohammadreza A. Determining the Level of Hospitalized Patients Satisfaction of Hospitals: A Systematic Review and Meta-Analysis. Hospital Journal. 2013;12(1).[Persian]
35. Hadian E TA. Tax Evasion and Its Determinants in the Iranian Economy (1971-2007). 2013; 18(121): 39-58.
36. Mozayeni A.H MN. Investigate the interactions between economic growth and environmental destruction on health. Journal of Environmental Science and Technology. 2012; 14(1): 17-30.
37. Almasi H MAB, Qoreishi R. The Organizational Factors Affecting VAT Evasion (Case Study of Tehran City VAT Directorate). Journal of Tax Research. 2012; 20(15): 37-50.
38. Abdollah Milani M ARN. Tax Evasion from the Underground Economy in Iran. Journal of Tax Research. 2012; 20(13): 141-68.
39. Amin Rashti N RM. Investigating the effect of VAT on income distribution in selected countries. Tax Reaserch Journal.2011;59(11):63-83.
40. Moghimi M SN, Danesh SH, Akbari Moghadam B, Daneshvar M. . The Survey of Welfare and Environmental Effects of Green Tax and Decline Subsidy on Fueles in Iran by Using a Computable General Equilibrium Model. Agricultural Economics and Development. 2011; 19(75): 79-108.
41. Najarzadeh R RM, Aghaei Khondabi M. Estimating the elasticity of demand for goods and services subject to consumption and sales tax in Iran. Journal of Tax Research. 2007; 5: 7-30.
42. Zehi N MS. A Study on Factors Affecting Tax Evasion (A Case Study of East Azerbaijan Province). Journal of tax research. 2011; 18(9): 25-60.
43. Siu E TAM. Linking health and finance ministries to improve taxes on unhealthy products. Bulletin of the World Health Organization. 2022; 100 (9): 570-7. http://dx.doi.org/10.2471/BLT.22.288104.

44. Miracolo A SM, Mills M, Kanavos P. Sin taxes and their effect on consumption, revenue generation and health improvement: a systematic literature review in Latin America. Health Policy and Planning. 2021. 36(5): 790-810. https://doi.org/10.1093/heapol/czaa168.
45. Hernández-F M CA, Colchero M.A. Taxes to Unhealthy Food and Beverages and Oral Health in Mexico: An Observational Study. Caries Research. 2021; 55(3): 183-92. https://doi.org/10.1159/000515223.
46. Tönnies T HC, Paprott R, Seidel-Jacobs E, Christa Scheidt-Nave C, Brinks R, Hoyer A. Estimating the impact of tax policy interventions on the projected number and prevalence of adults with type 2 diabetes in Germany between 2020 and 2040. BMJ Open Diabetes Research & Care. 2021; 9(1): e001813. https://doi.org/10.1136/bmjdrc-2020-001813.
47. Hofman K.J SN, Swart E.C, Popkin B.M, Wen Ng S. South Africa's Health Promotion Levy: Excise tax findings and equity potential. Obesity Reviews. 2021; 22(9): e13301. https://doi.org/10.1111/obr.13301.
48. Pedroza-Tobias A CE, Mialon M, Carriedo A.A. Food and beverage industry interference in science and policy: efforts to block soda tax implementation in Mexico and prevent international diffusion. British Medical Journal Global Health. 2021; 6(8): e005662. https://doi.org/10.1136/bmjgh-2021-005662.
49. Marinello S, Leider J, Powell LM. Employment impacts of the San Francisco sugar-sweetened beverage tax 2 years after implementation. Plos one. 2021;16(6):e0252094.
50. Sacks G KJ, Backholer K. Do taxes on unhealthy foods and beverages influence food purchases? Current Nutrition Report. 2021; 10(3): 179-87. https://doi.org/10.1007/s13668-021-00358-0.
51. Fichera E MT, Lopez-Valcarcel B.G, Roche D. How do consumers respond to "sin taxes"? New evidence from a tax on sugary drinks. Social Science & Medicine. 2021; 274(4): 113799. https://doi.org/10.1016/j.socscimed.2021.113799.
52. Li P LZ, Du H, Feng T, Zuo J. Do environmental taxes reduce air pollution? Evidence from fossil-fuel power plants in China. Journal of Environmental Management. 2021. 295: 113112. https://doi.org/10.1016/j.jenvman.2021.113112
53. Wang J ML, Renstrom T. Optimal sin taxes in the presence of income taxes and health care. Economics Letters. 2020; 186. https://doi.org/10.1016/j.econlet.2019.108767.
54. Elliott L.M DSL, Topp S.M. Health Taxes on Tobacco, Alcohol, Food and Drinks in Low- and Middle-Income Countries: A Scoping Review of Policy Content, Actors, Process and Context. International Journal of Health Policy and Management. 2022; 11(4): 414-28. https://doi.org/10.34172/ijhpm.2020.170.
55. Summan A SN, Birckmayer J, Blecher E, Chaloupka FJ, Laxminarayan R. The potential global gains in health and revenue from increased taxation of tobacco, alcohol and sugar- sweetened beverages: a modelling analysis. BMJ Global Health. 2020; 5(3): e002143. https://doi.org/10.1136/bmjgh-2019-002143.
56. Wolde-Rufael Y M-WE. Do environmental taxes and environmental stringency policies reduce CO2emissions? Evidence from 7 emerging economies. Environmental Science and Pollution Research. 2021; 28(18): 22392-408. https://doi.org/10.1007/s11356-020-11475-8.

57. Guo B WY, Feng Y, Liang c, Tang L, Yao X, Hu F. The effects of environmental tax reform on urban air pollution: A quasi-natural experiment based on the Environmental Protection Tax Law. Front Public Health. 2022; 10: 967524. https://doi.org/10.3389/fpubh.2022.967524
58. Wu DC SV, Gupta P, Essue BM, Luong L, Jha P. Impact of cigarette tax increase on health and financing outcomes in four Indian states. Gates Open Research. 2020; 4: 49. https://doi.org/10.12688/gatesopenres.13127.1.
59. Abdool Karim S KP, Hofman K. Industry strategies in the parliamentary process of adopting a sugar-sweetened beverage tax in South Africa: a systematic mapping. BMC Globalization and Health. 2020; 16(1): 116. https://doi.org/10.1186/s12992-020-00647-3
60. Kao K-E JAC, Ohinmaa A, Paulden M. The health and financial impacts of a sugary drink tax across different income groups in Canada. Economics and Human Biology. 2020; 38: 100869. https://doi.org/10.1016/j.ehb.2020.100869.
61. Javadinasab H, Masoudi Asl I, Vosoogh-Moghaddam A, Najafi B. Comparing selected countries using sin tax policy in sustainable health financing: Implications for developing countries. The International journal of health planning and management. 2020;35(1):68-78.
62. Luong L, Vu LH. Impacts of excise taxation on non-alcoholic beverage consumption in Vietnam. Sustainability. 2020; 12(3): 1092, Doi:org/10.3390/su12031092
63. Doble B JLFA, Finkelstein. The effect of implicit and explicit taxes on the purchasing of 'high-in-calorie' products: A randomized controlled trial. Economics & Human Biology. 2020; .
64. Smith E, Scarborough P, Rayner M, Briggs A.DM. Should we tax unhealthy food and drink? Proceedings of the Nutrition Society. 2018; 77(3): 314-20, Doi:org/10.1017/S0029665117004165
65. Jain V, Crosby L, Baker P, Chalkidou PK. Equity as a consideration in evaluations of health taxes: a systematic review. THE LANCET. 2019; 394: S8, Doi:org/10.1016/S0140-6736(19)32805-3.
66. Salgado J.C, Ng S.W. Understanding heterogeneity in price changes and firm responses to a national unhealthy food tax in Mexico. Food policy. 2019; 89: 101783, Doi:org/10.1016/j.foodpol.2019..101783
67. Griffith R, O'Connell M, Smith K. Tax design in the alcohol market. Journal of public economics. 2019; 172: 20-35, Doi: org/10.1016/j.jpubeco.2018.12.005.
68. Allcott H, Lockwood B.B, Taubinsky D. Regressive sin taxes, with an Application to the Optimal soda tax. The Quarterly Journal of Economics. 2019; 134(3): 1557-626, Doi:org/10.1093/qje/qjz017
69. Partyka O, Pajewska M, Krysińska-Pisarek M, Domosławska-Żylińska K, Bulira-Pawełczyk J, Czerw A. Sin tax as a public health tool-strengths and weaknesses of this economic solution. Przegld Epidemiologiczny. 2019; 73(4): 567-75, Doi:org/10.32394/pe.73.53
70. Kim K.H, Kang E, Yun Y.H. Public support for health taxes and media regulation of harmful products in South Korea. BMC Public Health. 2019; 19(1): 665, Doi:org/10.1186/s12889-019-7044-2
71. Chaloupka FJ, Powell LM, Warner KE. The Use of Excise Taxes to Reduce Tobacco, Alcohol, and Sugary Beverage Consumption. Annual Review of Public Health. 2019; 40: 187-201, Doi:org/10.1146/annurev-publhealth-040218-043816
72. Minh H.V, Duyen N.T, Ngan T.T, Ngoc N.B, Son D.T, Hai P.T. Potential health impacts of increasing the cigarette tax in Viet Nam. The International Journal of Tuberculosis and Lung Disease. 2018; 22(11): 1378-82, Doi:org/10.5588/ijtld.18.0161
73. Wesseh P.K, Lin B. Optimal carbon taxes for China and implications for power generation, welfare and the environment. Energy Policy. 2018; 118: 1-8, Doi:org/10.1016/j.enpol.2018.03.031
74. Shang C, Chaloupka F.J, Fong G.T, Gupta P.C, Pednekar M.S. The Association Between State Value-Added Taxes and Tobacco Use in India—Evidence From GATS and TCP India Survey. Nicotine &Tobacco Research. 2018; 20(11): 1344-52, Doi:org/10.1093/ntr/ntx184
75. Signal L.N, Watts C, Murphy C, Eyles H, Mhurchu C.N. Appetite for health-related food taxes: New Zealand stakeholder views. Health Promotion International. 2018; 33(5): 791-800, Doi:org/10.1093/heapro/dax019
76. Springmann M, Mason-D'Croz D, Robinson S, and et al. Health-motivated taxes on red and processed meat: A modelling study on optimal tax levels and associated health impacts. PLoS One. 2018; 13(11): e0204139, Doi:org/10.1371/journal.pone.0204139
77. Smith E, Scarborough P, Rayner M, Briggs A.DM. Should we tax unhealthy food and drink? Proceedings of the Nutrition Society. 2018; 77(3): 314-20, Doi:org/10.1017/S0029665117004165
78. Jensen JD SS. State-of-the-art for food taxes to promote public health. Proceedings of the Nutrition Society. 2018; 77(2): 100-5. https://doi.org/10.1017/S0029665117004050.
79. Du M TA, Erzse A, Hofman K.J. Sugar-Sweetened Beverage Taxes: Industry Response and Tactics. The Yale Journal of Biology and Medicine. 2018. 91(2): 185-90.
80. Tamir O, Cohen-Yogev T, Furman-Assaf S, Endevelt R. Taxation of sugar sweetened beverages and unhealthy foods: a qualitative study of key opinion leaders' views. Israel Journal of Health Policy Research. 2018; 7(1): 43, Doi: org/10.1186/s13584-018-0240-
81. Nomaguchi T, Cunich M, Zapata-Diomedi B, Veerman JL. The impact on productivity of a hypothetical tax on sugar-sweetened beverages. Health Policy. 2017;121(AKE6):715-25.
82. Pui K.L OJ. Economics and environmental implications of fuel efficiency improvement in Malaysia: A computable general equilibrium approach. Journal of cleaner production. 2017; 156: 459-69. https://doi.org/10.1016/j.jclepro.2017.04.067.
83. Wright A, Smith KE, Hellowell M. Policy lessons from health taxes: a systematic review of empirical studies. BMC Public Health. 2017; 17(1): 583, Doi:org/10.1186/s12889-017-4497-z.
84. Cobiac L.J TK, Veerman L, Blakely. Taxes and Subsidies for Improving Diet and Population Health in Australia: A Cost Effectiveness Modelling Study. PLOS Medicine. 2017; 14(2): e1002232. https://doi.org/10.1371/journal.pmed.1002232.
85. Silver L.D, Ng S.W, Ryan-Ibarra S. Changes in prices, sales, consumer spending, and beverage consumption one year after a tax on sugar-sweetened beverages in Berkeley, California, US: A before-and-after study. PLoS Medicine. 2017; 14(4): e1002283, Doi:org/10.1371/journal.pmed.1002283

86. Mccoy D, Chigudu S, Tillmann. Framing the tax and health nexus: a neglected aspect of public health concern. Health Econmics, Policy, and Law. 2017; 12(2): 179-94, Doi:org/10.1017/S174413311600044X
87. Manyema M, Veerman L.J, Tugendhaft A, Labadarios D, Hofman K. Modelling the potential impact of a sugar-sweetened beverage tax on stroke mortality, costs and health-adjusted life years in South Africa. BMC Public Health. 2016; 16(1): 405, Doi:org/10.1186/s12889-016-3085-y
88. Capacci S, Allais O, Bonnet C, Mazzocchi M. The impact of the French soda tax on prices, purchases and tastes: an ex post evaluation. PLOS ONE. 2019; 14(10): e0223196, Doi:org/10.1371/journal.pone.0223196
89. Hellowell M, Smith KE, Wright A. Hard to avoid but difficult to sustain: Scotland's innovative health tax on large retailers selling tobacco and alcohol. The Milbank Quarterly. 2016; 94(4): 800-31, Doi:org/10.1111/1468-0009.12200
90. Briggs ADM, Mytton OT, Elhussein A, Scarborough P. Econometric and comparative risk assessment scenario modelling of the proposed UK sugary drink tax on health. THE LANCET. 2016; 388: S10, Doi:org/10.1016/S0140-6736(16)32246-2.
91. Reeves A, Gourtsoyannis Y, Basu S, McCoy D, McKee M, Stuckler D. Financing universal health coverage—effects of alternative tax structures on public health systems: cross-national modelling in 89 low-income and middle-income countries. The Lancet. 2015; 386(9990): 274-80, Doi:org/10.1016/S0140-6736(15)60574-8
92. O'Hare B, Curtis M. Health spending, illicit financial flows and tax incentives in Malawi. Malawi Medical Journal. 2014; 26(4) 133-7.
93. Paes NL. Tax expensees and their impacts on performance in health and education. Ciência & Saúde Coletiva. 2014;19(4):1245.
94. Barry C.L, Niederdeppe J, Gollust S.E. Taxes on Sugar-Sweetened Beverages: Results from a 2011 National Public Opinion Survey. American Journal of Preventive Medicine. 2013. 44(2): 158-63, Doi:org/10.1016/j.amepre.2012.09.065
95. Chaloupka F.J YA, Fong G.T. Tobacco taxes as a tobacco control strategy. Tobacco Control. 2012; 21(2):172-80. 10.1136/tobaccocontrol-2011-050417
96. Powell LM, Chriqui JF, Khan T, Wada R, Chaloupka FJ. Assessing the potential effectiveness of food and beverage taxes and subsidies for improving public health: a systematic review of prices, demand and body weight outcomes. Obesity reviews. 2013; 14(2): 110-28, Doi:org/10.1111/obr.12002
97. Tiffin R, Arnoult M. The public health impacts of a fat tax. European Journal of Clinical Nutrition. 2011. 65(4): 427-33, Doi:org/10.1038/ejcn.2010.281
98. Ekins P, Summerton P, Thoung C, Lee D. A major environmental tax reform for the UK: results for the economy, employment and the environment. Environmental and Resource Economics. 2011; 50(3): 447-74, Doi:org/10.1007/s10640-011-9484-8
99. Miguel C.D, Manzano B. Gradual green tax reforms. Energy Economics. 2011; 33(1): 550-58, Doi:org/10.1016/j.eneco.2011.07.026.
100. Dissou Y, Eyland T. Carbon control policies, competitiveness, and border tax adjustments. Energy Economics. 2011; 33(3): 556-64, Doi:org/10.1016/j.eneco.2011.01.003.
101. Härkänen T, Kotakorpi K, Pietinen P, Pirttilä J, Reinivuo H, Suoniemi I. The welfare effects of health-based food tax policy. Food Policy. 2011; 49(1): 196-206 , Doi:org/10.1016/j.foodpol.2014.07.001.
102. Uadiale O.M FTO, Ogunleye J.O. An Empirical Study of the Relationship between Culture and Personal Income Tax Evasion in Nigeria. European Journal of Economics, Finance and Administrative Sciences. 2010; 20(20): 116-26.
103. Ščasný M, Píša V, Pollitt H, Chewpreecha U. Analyzing macroeconomic effects of environmental taxation in the czech republic with the econometric e3me model. Finance a Uver: Czech Journal of Economics & Finance. 2009;59(5).
104. DeCicca P, McLeod L. Cigarette taxes and older adult smoking: evidence from recent large tax increases. Journal of Health Economics. 2008; 27(4): 918-29, Doi:org/10.1016/j.jhealeco.2007.11.005
105. Glomma G, Kawaguchi D, Sepulveda F. Green taxes and double dividends in a dynamic economy. Journal of Policy Modeling. 2008; 30(1): 19-32, Doi:org/10.1016/j.jpolmod.2007.09.001.
106. Mayeres I VRD. Modelling the health related benefits of environmental policies and their feedback effects: a CGE analysis for the EU countries with GEM-E3. The Energy Journal. 2008; 29(1): 135-50.
107. Keen M L. The Value-Added Tax: Its Causes and Consequences. Fiscal Affairs Department, International Monetary Fund (IMF). 2007.
108. Mytton O, Gray A, Rayner M, Rutter H. Could targeted food taxes improve health? Journal of Epidemiology and Community Health. 2007. 61(8): 689-94, Doi:org/10.1136/jech.2006.047746
109. O'Donoghue T, Rabin M. Optimal sin taxes. Journal of Public Economics. 2006; 90(10-11): 1825-49, Doi:org/10.1016/j.jpubeco.2006.03.001.
110. Van Baal P.HM, Brouver W.BF, Hoogenveen R.T, Feenstra T.L. Increasing tobacco taxes: a cheap tool to increase public health. Health Policy. 2007; 82(2): 142-52, Doi:org/10.1016/j.healthpol.2006.09.004
